# Exposure to family stressful life events in autistic children: Longitudinal associations with mental health and the moderating role of cognitive flexibility

**DOI:** 10.1177/13623613211061932

**Published:** 2022-01-04

**Authors:** Virginia Carter Leno, Nicola Wright, Andrew Pickles, Rachael Bedford, Anat Zaidman-Zait, Connor Kerns, Pat Mirenda, Lonnie Zwaigenbaum, Eric Duku, Teresa Bennett, Stelios Georgiades, Isabel Smith, Tracy Vaillancourt, Peter Szatmari, Mayada Elsabbagh

**Affiliations:** 1 King’s College London, UK; 2 Bath University, UK; 3 Tel Aviv University, Israel; 4 The University of British Columbia, Canada; 5 University of Alberta, Canada; 6 Offord Centre for Child Studies, Canada; 7 McMaster University, Canada; 8 Dalhousie University, Canada; 9 IWK Health Centre, Canada; 10 University of Ottawa, Canada; 11 University of Toronto, Canada; 12 The Hospital for Sick Children, Canada; 13 McGill University, Canada

**Keywords:** autism spectrum disorder, cognitive flexibility, executive functioning, mental health, stressful life events

## Abstract

Mental health problems are prevalent in autistic youth, but the underpinning mechanisms are not well explored. In neurotypical youth, stressful life events are an established risk factor for mental health problems. This study tested longitudinal bidirectional associations between family-level stressful life events and mental health problems and whether these were moderated by cognitive flexibility, in a cohort of autistic children (*N* = 247). Family-stressful life events, assessed using the parent-reported Family Inventory of Life Events and Changes, and mental health problems, assessed using the teacher-reported Child Behavior Checklist Internalizing and Externalizing Symptoms subscales, were measured at multiple points between 7 and 11 years. Analyses showed no significant pathways from internalizing or externalizing symptoms to family-stressful life events or from family-stressful life events to internalizing or externalizing symptoms. There was some evidence of moderation by cognitive flexibility; the family-stressful life events to internalizing symptoms pathway was non-significant in the group with typical shifting ability but significant in the group with clinically significant shifting problems. Information about family-level stressful life event exposure and cognitive flexibility may be helpful in identifying autistic youth who may be at higher risk of developing mental health problems. Established risk factors for mental health problems in neurotypical populations are relevant for understanding mental health in autistic youth.

**Lay abstract:**

Experiencing stressful life events, such as a parent having had serious illness, parental divorce, bullying and victimization, is known to increase risk for mental health difficulties in neurotypical children. However, few studies have looked at whether stressful life events have a similar impact in autistic youth and if any individual characteristics may moderate the impact of said life events. In this study, we tested whether in autistic children aged 7–11 years, exposure to family-level stressful life events predicted later mental health symptoms (and vice versa). We also tested whether associations between stressful life events and mental health symptoms differed depending on the child’s level of cognitive flexibility. We found stressful life events only predicted internalizing symptoms (such as anxiety and depression) in children with clinically significant difficulties in cognitive flexibility (as rated by their parents). Mental health symptoms did not predict future exposure to stressful life events. Results suggest that information about exposure to stressful life events and cognitive inflexibility may be helpful in identifying autistic children who may be at risk of developing anxiety and depression symptoms.

## Introduction

Studies from typically developing children and adolescents suggest that experiencing stressful life events (SLEs; in this article, this term is used interchangeably with adverse life events) increases the likelihood of developing subsequent mental health problems (March-Llanes et al., 2017). Similar associations are found in children with intellectual disability (Hatton & Emerson, 2004). Although there is evidence to suggest that children and adolescents with autism spectrum disorder (henceforth referred to as autistic) may be more likely to experience SLEs (Green et al., 2004; Kerns et al., 2017), Hoover and Kaufman (2018) note that there is limited research exploring the association between SLEs and mental health problems in autistic youth (Ghaziuddin et al., 1995; Kerns et al., 2017; Taylor & Gotham, 2016). Given the increased prevalence of mental health problems in autistic individuals (Lai et al., 2019), advancing understanding of predictors of psychopathology in this population is an important step towards identifying those who may be at higher risk, providing appropriate interventions and promoting positive outcomes.

### SLEs and psychopathology in typically developing populations

Researchers have demonstrated that SLEs predict poorer mental health in typically developing children and adolescents (Grant et al., 2004; McLaughlin & Hatzenbuehler, 2009), when SLEs are conceptualized at either the family (e.g. family stress; Bøe et al., 2018) or child (e.g. bullying; Moore et al., 2017) level. Most work has focused on internalizing symptoms (i.e. anxiety and depression), although some evidence suggests a similar impact on externalizing symptoms (Kim et al., 2003; Tiet et al., 2001). Such effects may permeate beyond childhood, with studies reporting exposure to SLEs in childhood is associated with increased likelihood of psychopathology through late adolescence (Schilling et al., 2007) and adulthood (Chapman et al., 2004). Furthermore, bidirectional effects are reported; adolescents with higher levels of psychopathology are more likely to go on to experience SLEs (Grant et al., 2004; Kim et al., 2003), creating cycles of disadvantage.

While associations between SLEs and psychopathology are present at a group level, not all youth who experience SLEs develop mental health problems (Goodyer, 1993). A body of work has established the importance of moderating factors, providing evidence that environmental (e.g. supportive parenting; Flouri et al., 2015) and individual (e.g. general cognitive ability; Bridger & Daly, 2018) factors play a key role. One important individual characteristic thought to moderate associations between SLEs and internalizing problems is cognitive shifting ability (De Lissnyder et al., 2010; Hankin & Abramson, 2001; Stange et al., 2017). Cognitive shifting can be defined as an individual’s ability to shift to different thoughts or actions depending on situational demands (Monsell, 2003). Difficulties in cognitive flexibility are thought to impede emotional control and are linked to a ruminative response style (Davis & Nolen-Hoeksema, 2000; Hilt et al., 2014). In typically developing adults, rumination after SLE exposure predicts poorer outcomes and more mental health symptoms, even when adjusting for severity of pre-SLE symptoms (Ruscio et al., 2015), and emotional control and cognitive shifting moderate the association between exposure to community violence and anxiety symptoms in children (Burgers & Drabick, 2016).

### Family-SLEs and psychopathology in autistic populations

Despite the established link between SLEs and psychopathology in neurotypical youth, the high rates of mental health problems in autistic youth (Simonoff et al., 2008) and evidence to suggest that autistic children may be more likely to experience individual SLEs than children without an autism diagnosis (Green et al., 2004; Kerns et al., 2017), few studies have examined the association between SLEs and mental health in autistic individuals (see Hoover & Kaufman, 2018, for a review). Some studies report cross-sectional association between SLEs and internalizing symptoms in autistic children (Ghaziuddin et al., 1995; Kerns et al., 2017, 2020) and older adolescents (Taylor & Gotham, 2016). Similar associations have also been reported with externalizing problems in autistic children (Brenner et al., 2018; McDonnell et al., 2019). However, to our knowledge, researchers have not tested whether exposure to SLEs predicts later psychopathology in autistic youth. Testing for longitudinal association is a crucial step in establishing causal links between SLEs and mental health problems. This has important clinical implications regarding how information about SLEs could be used to inform the likelihood of subsequent mental health problems in autistic youth.

The potential moderating role of cognitive shifting is especially pertinent in autistic individuals, who are known to have particular difficulties in this domain (Landry & Al-Taie, 2016). These difficulties, thought to underpin the repetitive patterns of thoughts and behaviour often experienced by autistic individuals (Miller et al., 2015), may also increase the risk of mental health problems following SLEs (Kerns et al., 2015). As proposed in typically developing individuals, impairments in cognitive flexibility may mean that autistic youth especially struggle with disengagement from memories of distressing stimuli, leading to increased rumination and consequent mental health problems.

### Aims

We used a large *N* longitudinal study of autistic children to test pathways between family exposure to family-level SLEs (henceforth referred to as family-SLEs) and child mental health problems and whether cognitive shifting ability moderates the pathway from family-SLE exposure to child mental health problems. We predicted bidirectional pathways between family-SLEs and psychopathology, with cognitive flexibility moderating the pathway from family-SLE to psychopathology (in that the impact of family-SLEs on future mental health problems would be significantly stronger in autistic individuals with difficulties in cognitive flexibility).

## Method

### Participants

Data for this study were drawn from the Pathways in Autism Spectrum Disorder study, a prospective, longitudinal cohort study examining developmental trajectories of autistic children (*n* = 421). The Pathways sample is a large inception cohort of autistic children, recruited at time of diagnosis from five sites across Canada starting in 2005. Inclusion criteria upon entry to the study were (a) age between 2 and 5 years at enrolment and (b) a recent diagnosis of autism spectrum disorder (<4 months prior to enrolment). Diagnosis was confirmed using *Diagnostic and Statistical Manual of Mental Disorders* (4th ed., text rev.; DSM-IV-TR) criteria and both the Autism Diagnostic Observation Schedule (ADOS; Lord et al., 2002) and the Autism Diagnostic Inventory–Revised (ADI-R; Rutter et al., 2003). Children with a diagnosis of cerebral palsy or other neuromotor disorders, identified genetic or chromosomal abnormalities, or significantly impaired vision or hearing were excluded. Caregivers were required to be verbally proficient in English (or French, in Quebec). Assessment occurred at baseline (T1: mean age = 3.46 years), approximately 6 and 12 months after baseline (T2: mean age = 3.99 years, and T3: mean age = 4.51 years), at age 6 (T4: mean age = 6.66 years), and then at four time points approximately 1 year apart (T5 to T8: mean ages = 7.77 years, 8.73 years, 9.71 years, 10.77 years, respectively).

### Missing data

Between recruitment and T4, *N* = 103 families had withdrawn from the study, resulting in *N* = 318 approached for T5. In this study, participants were included who had at least one measurement of family-SLEs or mental health problems at T5, T6, T7 or T8, resulting in 247 participants for primary analyses. *N* = 155 had complete data on family-SLEs and mental health problems at T5 and T6, *N* = 114 had complete data at T5, T6 and T7 and *N* = 95 had complete data at T5, T6, T7 and T8. In all, 90.3% of participants had two or more measurements of family-SLEs or mental health problems. Attrition analyses found that the sample who had complete data across all four waves did not differ on T5 family-SLEs, T5 mental health problems, T5 cognitive flexibility or T4 severity of autism symptoms as compared with participants who did not complete the full four waves of assessments (*p* = 0.66 for family-SLEs, *p* = 0.98 for internalizing problems, *p* = 0.95 for externalizing problems, *p* = 0.89 for shifting *T* score, *p* = 0.19 for autism symptoms). Differences were found in T4 IQ, in that those who completed all waves had higher IQ as compared with those dropped out between T5 and T8 (mean IQ = 82.62 (standard deviation (*SD*) = 17.55) for sample that dropped out, mean IQ = 88.16 (*SD* = 20.48) for sample with complete data, *p* = 0.05).

### Measures

#### Family-SLEs

Data on the parent-reported Family Inventory of Life Events and Changes (FILE; McCubbin et al., 1996) were collected at T5, T6, T7 and T8. This instrument assesses whether 71 normative and non-normative SLEs have been experienced by the family unit during the previous 12 months. The total score is formed of the sum of nine subscales: Intra-family Strains, Marital Strains, Pregnancy and Childbearing Strains, Finance and Business Strains, Work–Family Transition Strains, Illness and Family Care Strains, Losses, Transitions and Family Legal Violations. Internal consistency was good to excellent in the current sample (ranging from α = 0.85–0.95 across T5–T8).

#### Mental health problems

Data on the Teacher-Report Form of the Child Behavior Checklist (6–18 years version; CBCL; Achenbach & Rescorla, 2001) were collected at T5, T6, T7 and T8 to measure the child’s mental health symptoms over the previous 6 months. We chose to use the teacher-report version to avoid common method variance with parent-reported family-SLEs. Current analyses used the Internalizing and Externalizing Symptoms subscales’ total raw scores to measure the symptoms of emotional and behavioural disorders, respectively. These subscales are derived from five syndrome scales (the Internalizing subscale consists of items from the Anxious/Depressed, Withdrawn/Depressed and Somatic Complaints subscales; the Externalizing subscale consists of items from the Rule Breaking Behavior and Aggressive Behavior subscales). The psychometric properties of the CBCL in autistic populations have been examined elsewhere and found to be comparable with that reported in typically developing samples (Pandolfi et al., 2012).

#### Cognitive flexibility

Parent-report on the Behaviour Inventory Rating of Executive Function (BRIEF; Gioia et al., 2000) was used at T5 (and T6 where T5 data were unavailable (*n* = 36); based on the observation that the correlation between T5 and T6 scores was high, *r* = 0.72, *p* < 0.01). The BRIEF is designed to measure executive functioning (EF) in real world settings over the prior 6 months in children aged 5–18 years. Analyses focused on the Shifting subscale, which assesses a child’s ability to ‘move freely from one situation, activity, or aspect of a problem to another as the situation demands; transition, solve problems flexibly’ (Gioia et al., 2000), with a higher score indicating more problems in this domain. Autistic youth are distinguished from other clinical groups by their lower scores on the Shift subscale (Gioia et al., 2002), meaning it is often conceptualized as a measure of cognitive inflexibility. To test for moderation of pathways by cognitive flexibility ability, individuals with *T* score of ⩾65, indicating clinically significant difficulties, were designated as the clinically significant shifting problems group (*n* = 98), whereas those scoring below cut-off were designated as the typical shifting ability group (*n* = 148; Gioia et al., 2000). Participants who were missing BRIEF data at T5 and T6 could not be classified and therefore were not included in moderation analyses (*n* = 49). Internal consistency of the Shift subscale was good in the current sample (α = 0.83).

#### Family income

Parents were asked to indicate family income at T5 along an 11-point scale (1 = <$5000 CAD to 11 = >$80,000 CAD).

#### IQ

Child IQ was measured at T6 using the Wechsler Intelligence Scale for Children, 4th edition (WISC-IV; Wechsler et al., 2004).

#### Autism symptomatology

Autism symptomatology was measured at T4 using the ADOS (Lord et al., 2002) calibrated severity score, with a higher score indicative of a higher level of autism symptoms.

### Statistical analysis

**Figure 1.**
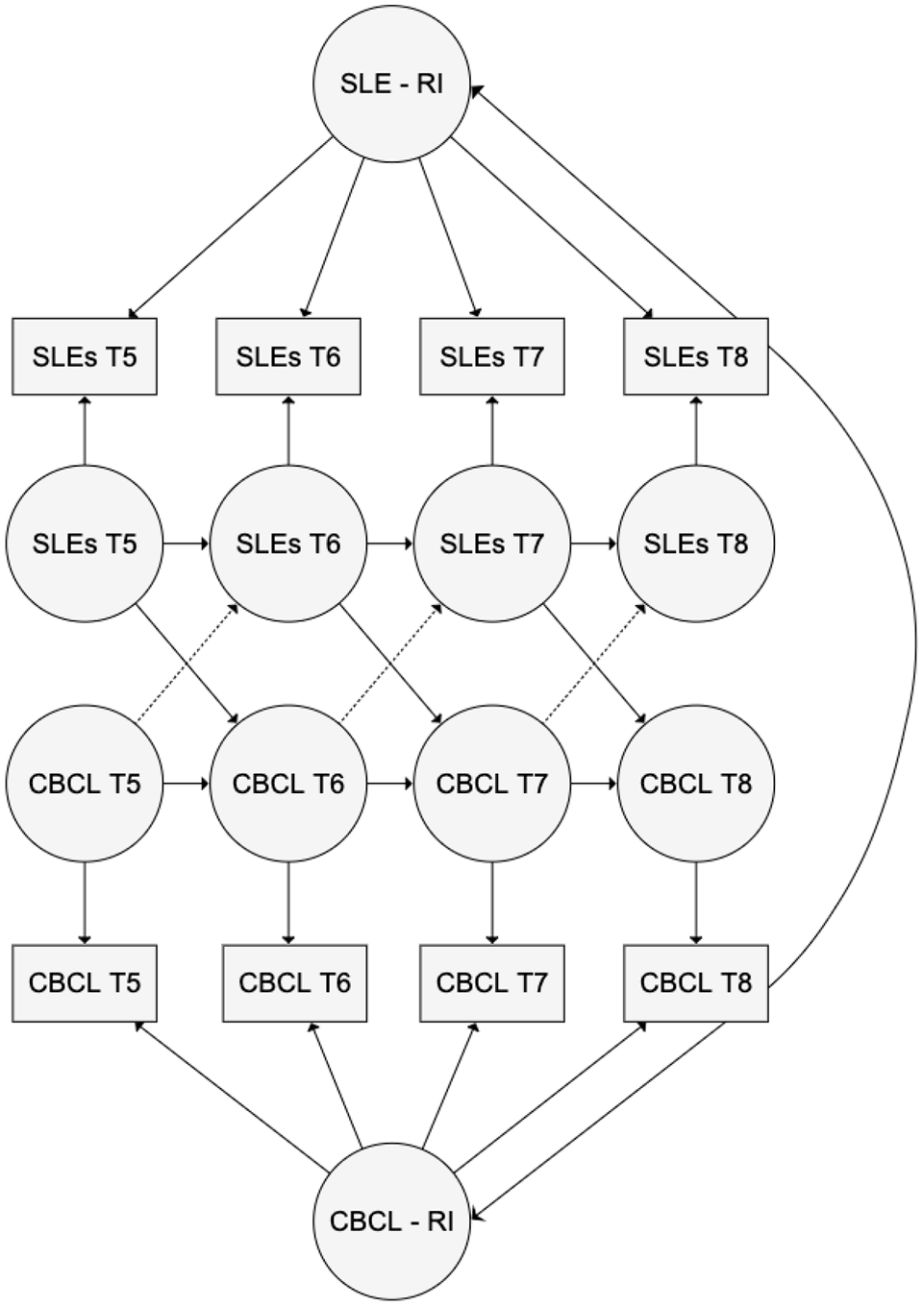
Random intercept (RI)–cross-lag path model testing associations between family-level stressful life events (SLEs) and mental health problems as measured by the Child Behavior Checklist (CBCL).

Descriptive statistics and bivariate correlations were calculated in Stata 14 (StataCorp, 2009). Due to skewed distributions, scores from the FILE and CBCL were square-root-transformed. To test bidirectional associations between exposure to family-SLEs and mental health problems, we used random intercept–cross-lag panel models (RI-CLPM, an extension of traditional cross-lagged panel models), which account for time-invariant, trait-like between-individual differences through the inclusion of a random intercept (Hamaker et al., 2015). By partitioning between-individual differences and within-individual change, this model allowed estimation of the extent to which within-person change in exposure to family-SLEs predicts within-person change in mental health problems and vice versa. We regressed the observed score at each time point for family-SLEs and CBCL internalizing and externalizing symptoms (two models were run, one for Internalizing and one for Externalizing subscales) onto independent latent factors, constraining the loading to be the same across time points for each measure. We specified auto-regressive pathways between the factors, cross-sectional correlations at each time point and cross-lagged pathways in both directions; however, as our prediction primarily focused on testing the predictive effect of family-SLEs on later psychopathology, if the opposite pathways (the dotted cross-lag paths in Figure 1) were non-significant, they were dropped from the model. Auto-regressive, cross-sectional correlations and cross-lag pathways were constrained to be the same across all time points as we did not have any hypotheses about age-specific effects. To account for between-individual differences, we regressed the observed family-SLEs and CBCL scores onto two overarching random intercept factors (family-SLE-RI and CBCL-RI in Figure 1), where all loadings were set to one, and allowed these two factors to correlate. RI-CLPM have numerous latent variables, covariances and error variances often resulting in some infeasible estimates when unconstrained. Such infeasible estimates (e.g. negative variances) were set to zero. The variance of the family-SLE-RI factor was set to zero, with the assumption being that there would not be strong trait-like differences in an environmentally driven variable such as parent-reported family-SLEs beyond those accounted for by the auto-regressive pathways. Once we had tested the pathways of interest in the full model, we ran a multi-group model using the two pre-defined groups (typical shifting ability vs clinically significant shifting problems) and compared the coefficient for the family-SLEs→CBCL pathway between the two groups using the Wald test of parameter constraints. For structural equation analyses, we report both unstandardized (*b*) and standardized (β) coefficients. All models were estimated in Mplus 7 (Muthén & Muthén, 1998–2012) using full maximum likelihood with robust standard errors to account for missing data. Model fit was indicated by the χ^2^ statistic, comparative fit index/Tucker–Lewis index (CFI/TLI) and root mean square error of approximation (RMSEA). Bivariate correlations between family-SLEs and CBCL internalizing and externalizing symptoms for the whole sample are presented in Table S1 and for typical shifting ability versus significant shifting problems groups in Tables S2 and S3. Although the inclusion of a random intercept factor adjusts cross-lag pathways for any time-invariant individual differences, sensitivity analyses first re-ran models with (1) T5 income and then (2) T4 ADOS calibrated severity scores regressed on the family-SLE-RI and CBCL-RI factors to check that differences in income or autism symptomatology were not driving any significant results (income data available from *n* = 190, ADOS data available from *n* = 241 of the primary *N* = 247 sample). Second, to better understand the specificity of any effects to cognitive flexibility, we re-ran multi-group models with T6 IQ as the moderator instead of BRIEF shift scores. A binary variable was created by dividing participants into two groups; those with IQ ⩽ 80 (*n* = 70) and those with IQ > 80 (*n* = 110) (*n* = 60 from the primary *N* = 247 sample were missing T6 IQ data so were not included in this sensitivity analysis). At reviewers’ suggestion, we also ran additional post hoc models using the parent-report CBCL to measure mental health problems to examine the generalizability of effects (see Supplementary Materials).

### Community involvement

The aims and objectives of the Pathways in Autism Spectrum Disorder study were determined by a meeting of parents, advocates, practitioners and researchers in 2005 (www.asdpathways.ca). Community members have been engaged in aspects of the study over the years.

## Results

Table 1 presents sample demographic information and summary statistics for all variables included in analysis. Table 2 presents study variables split by typical shifting ability versus clinically significant shifting problems grouping variable.

**Table 1.** Demographic characteristics and key variables.

	Mean (standard deviation)	*N* available data in current analyses
Median age of diagnosis (years)	3.36 (interquartile interval Q1–Q3: 2.81–3.94)	n/a
Sex (% girls)	15.8	n/a
Mother ethnicity (% White)	74.2	n/a
Child IQ T6 (WISC)	85.51 (18.74)	187
Median income category T5	9 (interquartile interval Q1–Q3: 7–11)	190
FILE total T5	8.87 (6.89)	194
FILE total T6	8.19 (6.46)	204
FILE total T7	6.97 (6.22)	150
FILE total T8	7.15 (5.24)	163
CBCL internalizing T5	8.45 (5.81)	151
CBCL internalizing T6	8.24 (6.27)	144
CBCL internalizing T7	10.01 (6.91)	128
CBCL internalizing T8	8.96 (6.24)	123
CBCL externalizing T5	11.25 (8.48)	151
CBCL externalizing T6	9.29 (8.85)	144
CBCL externalizing T7	9.38 (8.30)	128
CBCL externalizing T8	8.97 (8.18)	123
BRIEF shifting *T* score T5	62.13 (12.89)	198
BRIEF T5 age of assessment (years)	7.75 (0.22)	n/a

n/a: not applicable as variable not used in current analyses; WISC: Wechsler Intelligence Scale for Children; FILE: Family Inventory of Life Events and Changes; CBCL: Child Behavior Checklist; BRIEF: Behaviour Inventory Rating of Executive Function.

**Table 2.** Comparison of key variables by shifting group status.

Mean (standard deviation)	Typical shifting ability (*n* = 144)	Clinically significant shifting problems (*n* = 98)	*t* test of group differences
FILE total T5	7.13 (5.77)	11.41 (7.59)	*p* < 0.01
FILE total T6	6.50 (5.41)	10.65 (7.07)	*p* < 0.01
FILE total T7	5.32 (4.78)	9.32 (7.23)	*p* < 0.01
FILE total T8	6.11 (4.89)	8.79 (5.40)	*p* < 0.01
CBCL internalizing T5	7.73 (5.38)	9.38 (6.24)	*p* = 0.14
CBCL internalizing T6	8.19 (5.81)	8.30 (6.86)	*p* = 0.80
CBCL internalizing T7	9.71 (6.49)	10.43 (7.51)	*p* = 0.71
CBCL internalizing T8	8.35 (6.49)	9.82 (5.82)	*p* = 0.14
CBCL externalizing T5	10.31 (8.29)	12.45 (8.64)	*p* = 0.07
CBCL externalizing T6	7.85 (7.71)	11.14 (9.89)	*p* = 0.08
CBCL externalizing T7	8.36 (7.82)	10.83 (8.81)	*p* = 0.07
CBCL externalizing T8	8.58 (8.83)	9.51 (7.21)	*p* = 0.28
BRIEF shifting *T* score T5	53.69 (7.85)	74.59 (7.72)	*p* < 0.01
BRIEF T5 age of assessment (years)	7.76 (0.22)	7.73 (0.22)	*p* = 0.32
Sex (% girls)	11%	27%	χ^2^ < 0.01
T4 autism symptoms (ADOS-CSS)	6.72 (2.18)	7.58 (1.94)	*p* < 0.01
T6 IQ (WISC)	86.55 (18.96)	82.70 (19.21)	*p* *=* *0.32*

FILE: Family Inventory of Life Events and Changes; CBCL: Child Behavior Checklist; BRIEF: Behaviour Inventory Rating of Executive Function; ADOS-CSS: Autism Diagnostic Observation Schedule–Calibrated Severity Score; WISC: Wechsler Intelligence Scale for Children.

The table presents untransformed values, but *t* tests were run on transformed scores.

### Internalizing symptoms

In the full sample, the pathway (at all time points) from internalizing symptoms to family-SLEs was non-significant (*b* = –0.02, 95% confidence intervals (CIs) = (–0.13, 0.09); β = –0.04, 95% CIs = (–0.22, 0.14); *p* = 0.74) and therefore dropped from the model. The pathway from family-SLEs to internalizing symptoms was non-significant (*b* = 0.12, 95% CIs = (0.02, 0.23); β = 0.14, 95% CIs = (0.01, 0.27); *p* = 0.06). Auto-regressive pathways (indicating within-domain longitudinal prediction) for family-SLEs (*b* = 0.90, 95% CIs = (0.84, 0.97); β = 0.96, 95% CIs = (0.89, 1.03); *p* < 0.01) and internalizing symptoms (*b* = 0.74, 95% CIs = (0.32, 1.17); β = 0.69, 95% CIs = (0.31, 1.06); *p* < 0.01) were both significant. Cross-sectional correlations between family-SLEs and internalizing symptoms were non-significant (*b* = 0.05, 95% CIs = (–0.05, 0.15); β = 0.07, 95% CIs = (–0.07, 0.21); *p* = 0.41). Model fit was excellent (χ^2^(24) = 16.88, *p* = 0.85, CFI/TLI = 1.00, RMSEA = 0.00).

**Figure 2.**
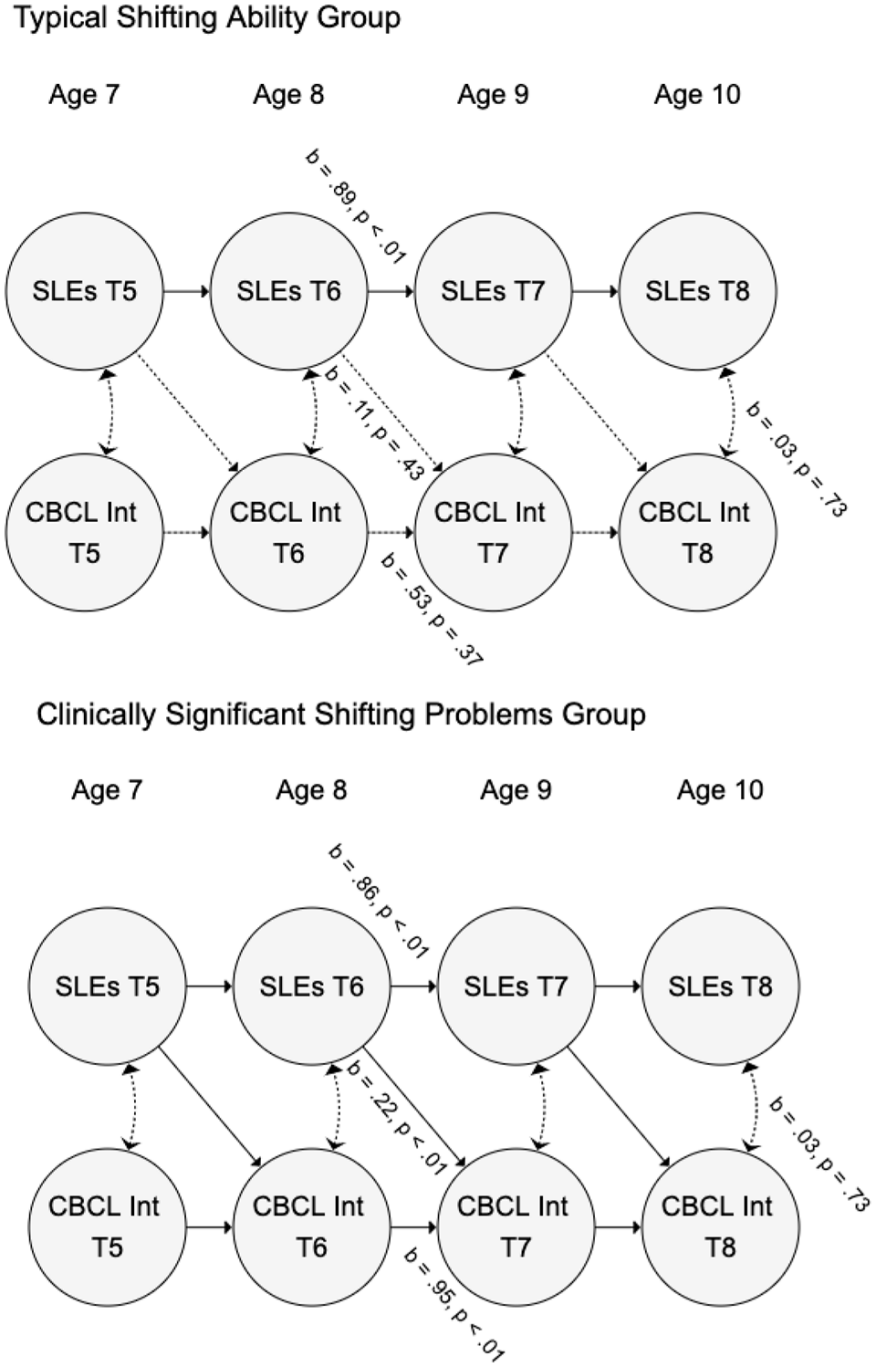
Moderation of associations between family-level stressful life events (SLEs) and internalizing problems as measured by the Child Behavior Checklist (CBCL Int) by shifting ability in autistic youth. Auto-regressive, cross-lag and correlational paths were fixed to be equivalent at each time point, so only one parameter is given for each. Random intercepts and observed variables are omitted for clarity.

When the sample was split by level of shifting problems (see Figure 2), the pathway from family-SLEs to internalizing symptoms was non-significant in the typical shifting group (*b* = 0.11, 95% CIs = (–0.12, 0.34); β = 0.11, 95% CIs = (–0.11, 0.33); *p* = 0.43) but significant in the clinically significant shifting problems group (*b* = 0.22, 95% CIs = (0.08, 0.35); β = 0.20, 95% CIs = (0.07, 0.32); *p* < 0.01). The auto-regressive pathway for family-SLEs was significant in both the typical shifting and clinically significant shifting problems groups (*b* = 0.89, 95% CIs = (0.81, 0.94); β = 0.95, 95% CIs = (0.87, 1.04); *p* < 0.01; *b* = 0.86, 95% CIs = (0.72, 1.00); β = 0.92, 95% CIs = (0.79, 1.05); *p* < 0.01, respectively). The auto-regressive pathway for internalizing symptoms was non-significant in the typical shifting group (*b* = 0.53, 95% CIs = (–0.44, 1.50); β = 0.49, 95% CIs = (–0.35, 1.32); *p* = 0.37) but significant in the clinically significant shifting problems group (*b* = 0.95, 95% CIs = (0.61, 1.28); β = 0.95, 95% CIs = (0.60, 1.29); *p* < 0.01). Cross-sectional correlations between family-SLEs and internalizing symptoms were non-significant in both groups (*b* = 0.03, 95% CIs = (–0.12, 0.18); β = 0.05, 95% CIs = (–0.18, 0.27); *p* = 0.73 for both). The Wald test of group differences in the family-SLEs to internalizing symptoms path was non-significant (*p* = 0.50).

### Externalizing symptoms

In the full sample, the pathway from externalizing symptoms to family-SLEs was non-significant and therefore dropped from the model (*b* = –0.02, 95% CIs = (–0.14, 0.09); β = –0.04, 95% CIs = (–0.22, 0.14); *p* = 0.74). The pathway from family-SLEs to externalizing symptoms was also non-significant (*b* = 0.10, 95% CIs = (–0.08, 0.27); β = 0.06, 95% CIs = (–0.05, 0.17); *p* = 0.37). The auto-regressive pathway was significant for family-SLEs (*b* = 0.91, 95% CIs = (0.83, 0.98); β = 0.95, 95% CIs = (0.89, 0.04); *p* < 0.01) but not externalizing symptoms (*b* = 0.26, 95% CIs = (–0.01, 0.52); β = 0.25, 95% CIs = (0.01, 0.50); *p* = 0.10). Cross-sectional correlations between family-SLEs and externalizing symptoms were significant (*b* = 0.15, 95% CIs = (0.05, 0.25); β = 0.12, 95% CIs = (0.03, 0.22); *p* = 0.02). Model fit was excellent (χ^2^(24) = 20.76, *p* = 0.65, CFI/TLI = 1.00, RMSEA = 0.00).

**Figure 3.**
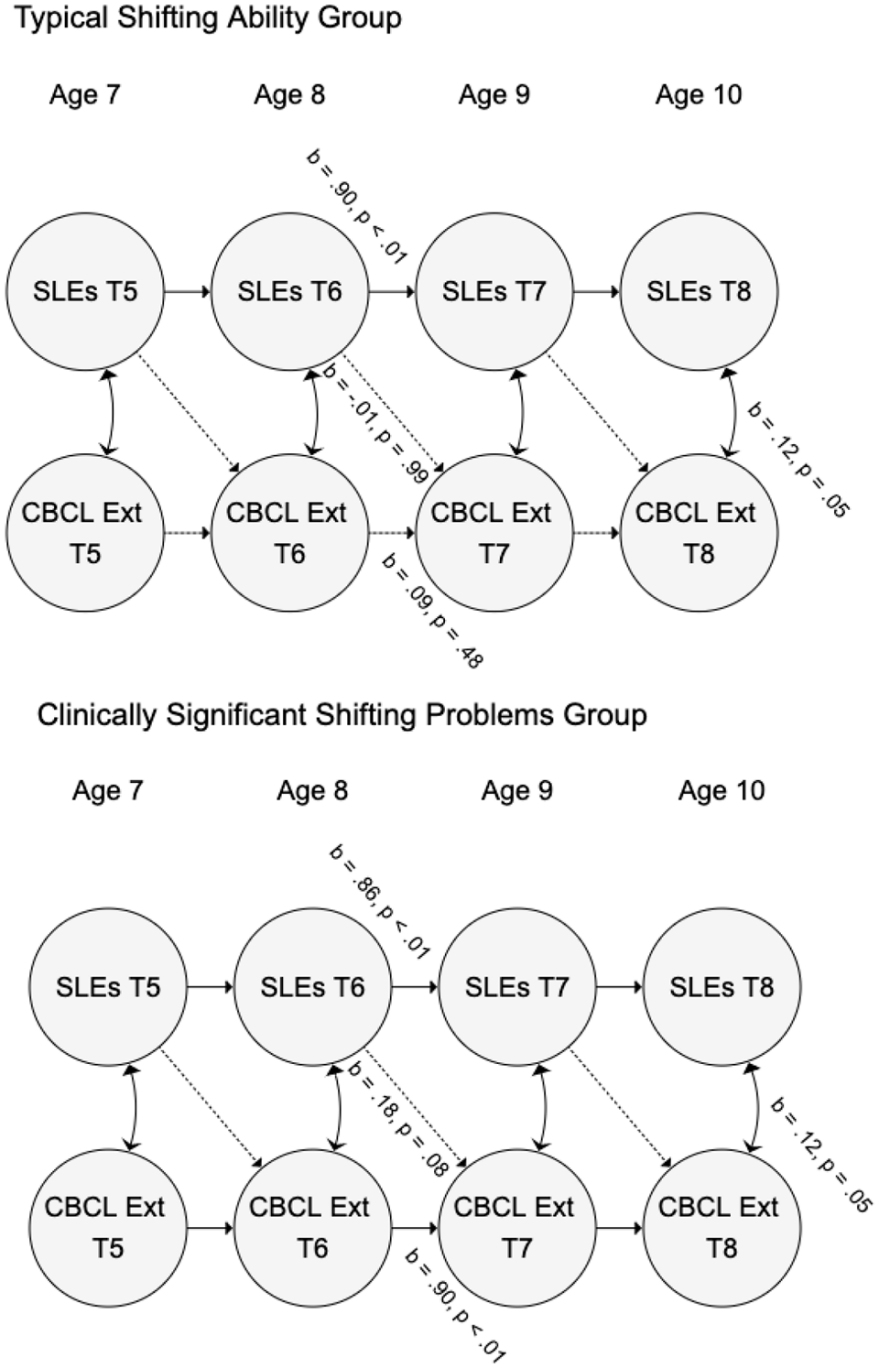
Moderation of associations between family-level stressful life events (SLEs) and externalizing problems as measured by the Child Behavior Checklist (CBCL Int) by shifting ability in autistic youth. Auto-regressive, cross-lag and correlational paths were fixed to be equivalent at each time point, so only one parameter is given for each. Random intercepts and observed variables are omitted for clarity.

When the sample was split by BRIEF shifting problems (see Figure 3), the pathway from family-SLEs to externalizing symptoms remained non-significant in both typical shifting (*b* = –0.01, 95% CIs = (–0.25, 0.24); β = –0.02, 95% CIs = (–0.16, 0.16); *p* = 0.99) and clinically significant shifting problems groups (*b* = 0.18, 95% CIs = (0.01, 0.35); β = 0.11, 95% CIs = (0.01, 0.21); *p* = 0.08). The auto-regressive pathway for family-SLEs was significant in both groups (*b* = 0.90, 95% CIs = (0.81, 0.98); β = 0.96, 95% CIs = (0.87, 1.04); *p* < 0.01; *b* = 0.86, 95% CIs = (0.72, 0.99); β = 0.92, 95% CIs = (0.79, 1.05); *p* < 0.01, respectively). The auto-regressive pathway for externalizing symptoms was non-significant in the typical shifting group (*b* = 0.09, 95% CIs = (–0.10, 0.27); β = 0.09, 95% CIs = (–0.01, 0.27); *p* = 0.48) but significant in the clinically significant shifting problems group (*b* = 0.90, 95% CIs = (0.74, 1.06); β = 0.90, 95% CIs = (0.73, 1.06); *p* < 0.01). Cross-sectional correlations between family-SLEs and externalizing symptoms were at significance in both groups (*b* = 0.12, 95% CIs = (0.02, 0.23); β = 0.11, 95% CIs = (0.02, 0.22); *p* = 0.05; *b* = 0.12, 95% CIs = (0.02, 0.23); β = 0.08, 95% CIs = (0.02, 0.15); *p* = 0.05, respectively). The Wald test of group differences in the family-SLEs to externalizing symptoms path was non-significant (*p* = 0.32).

### Sensitivity analyses

In models adjusting for income and autism symptomatology, the overall pattern of results remained largely unchanged, the pathway from family-SLEs internalizing symptoms in the clinically significant shifting problems group remained significant (*b* = 0.22, *p* < 0.01 when adjusting for T5 income, *b* = 0.21, *p* < 0.01 when adjusting for T4 autism symptomatology) and the pathway in the typical shifting group remained non-significant (*p*s > 0.32). In models using IQ rather than shifting ability as the moderator, all paths from family-SLEs to internalizing and externalizing were non-significant (*p*s = 0.14–0.94).

## Discussion

In this article, we tested bidirectional pathways between family-level SLEs and mental health problems in autistic children and whether individual differences in cognitive flexibility moderated the family-SLE to mental health problems pathway. The pathways from mental health problems to family-SLEs and from family-SLEs to mental health problems were both non-significant. Consistent with our prediction of moderation by cognitive flexibility, a significant pathway from family-SLEs to future internalizing problems was found in the group with clinically significant shifting problems but not in the group with typical shifting ability. A similar, albeit non-significant pattern was found for the prediction of externalizing problems. Furthermore, sensitivity analyses suggested that moderation by cognitive flexibility was relatively specific as the pattern of effects differed when IQ was used as the moderator of associations between family-SLEs and mental health problems, and adjusting for income and autism symptomatology did not change the pattern of results. Results suggest that both family-SLE exposure and cognitive flexibility should be considered when assessing autistic young people with mental health problems and may be potential targets for intervention.

We found that neither internalizing nor externalizing symptoms predicted future family-SLE exposure. In typically developing adolescents, higher levels of emotional and behavioural problems predict increased likelihood of experiencing SLEs (Grant et al., 2004; Kim et al., 2003). This is thought to be due, at least in part, to a person’s individual characteristics and behaviours eliciting SLEs, and therefore creating cycles of maladaptive process. There are multiple explanations for the current lack of reciprocal associations. First, the measure of SLEs used in this study asked about events that were happening to the whole family (e.g. strains due to marital, financial or health issues), which may be less amenable to influence by child psychopathology. SLEs more proximal to the child in question (e.g. bullying), or those reported by the child themselves, may be more likely to be predicted by child mental health. Relatedly, it is also possible that our sample was too young (7–11 years) to have the level of independence that is required to seek out or elicit certain environmental events (e.g. placing oneself in social circumstances increase the likelihood of experiencing SLEs). Studies that report reciprocal effects have used older samples (12–18 years in Kim et al., 2003), and the authors noted differential age effects, in that the effect of externalizing problems on SLEs was greater in late adolescence. Second, in the current sample, all had a diagnosis of autism. It may be that the parents of autistic children are more planful of their activities, leaving fewer opportunities for the child to influence their environment or the reverse. Third, the relative stability of family-SLEs at this age may have decreased our power to detect predictive associations.

In terms of effects on family-SLEs on mental health in the full sample, the impact of family-SLEs was not statistically significant (although *p* = 0.06 for the pathway from family-SLE to internalizing symptoms). This may have been in part due to our stringent statistical approach, which controlled for between-individual differences in mental health problems, something most cross-lag models do not consider. Furthermore, effects may have been larger in magnitude if SLEs measured were experienced by the child rather than the whole family. We also highlight that the sample was relatively young, with the final wave of data collection being at age 11. Given that adolescence is a key time for the emergence of mental health symptoms, and grants children more autonomy over the types of environments they experience, we might expect to see stronger effects as children get older. However, the direction of results is in line with previous cross-sectional studies that have found associations between SLEs and higher anxiety and depression in autistic youth (Ghaziuddin et al., 1995; Kerns et al., 2017, in press; Taylor & Gotham, 2016).

When the sample was split by shifting ability, family-SLEs significantly predicted internalizing symptoms in the group with clinically significant shifting problems (indicative of difficulties in cognitive flexibility) but not the group with typical cognitive shifting ability. The difference in coefficients for the family-SLE to internalizing symptoms pathway (β = 0.11 in the typical shifting group vs β = 0.22 in the shifting problems group) is consistent with our hypothesis that cognitive flexibility difficulties would moderate the impact of family-SLEs. However, the between-group test of coefficients was not significant; we suggest this may have been due to limited statistical power to robustly test moderation effects. Statistical models which can account for between-person variability while testing cross-lagged pathways and continuous moderators would be of use. Results are in line with typical developmental findings, where good EF buffers the impact of sub-optimal parenting on internalizing problems in school-age children (Gueron-Sela et al., 2018; Muhtadie et al., 2013). In adults, poorer cognitive flexibility is proposed to be a risk factor for internalizing disorders (especially depression) through the mechanism of increased rumination (De Lissnyder et al., 2010; Stange et al., 2017). Further research with more precise measurement of cognitive shifting abilities, in addition to assessment of rumination, would help to understand if the same mechanism is present in autistic youth.

Although we found no significant pathways from family-SLEs to externalizing symptoms in either group, we note that the pattern of effects is similar to that for internalizing symptoms, in that the pathway from family-SLEs to externalizing symptoms was much stronger in the group with clinically significant shifting problems (β = 0.11, *p* = 0.08, as compared with β = –0.01, *p* = 0.99 in the typical shifting ability group). However, the effect of family-SLEs on externalizing symptoms was clearly not as strong as the effect on internalizing symptoms. The lack of within-person variation (i.e. waxing and waning of symptoms) in externalizing problems over time may in part explain this. There was little variance in externalizing problems left to predict, once between-person stable differences were accounted for, as indicated by non-significant auto-regressive pathways. This is most likely due to the random intercept factor (conceptualized as accounting for trait-like between-person differences) accounting for all between-time correlations. The finding that stability in externalizing symptoms in this study was mostly explained by trait-like stability is consistent with findings from prior twin studies over this age range. Greater stability is reported for externalizing as compared with internalizing symptoms (Bartels et al., 2004), suggested to be largely the result of time-invariant genetic influences (Haberstick et al., 2005). However, we did find cross-sectional correlations between family-SLEs and externalizing behaviour. The FILE measure asks about SLEs that have occurred during the previous year, and the CBCL asks about behaviour that has occurred in the previous 6 months, meaning that some SLEs could precede externalizing behaviour whereas others overlap. Thus, more immediate effects from family-SLEs to externalizing behaviour remain possible in autistic youth. Alternatively, there may be some unmeasured third factor that could explain a simultaneous increase in family-SLE exposure and externalizing problems (e.g. a decrease in parental mental health). Answering this question requires more precise information about the timing of SLEs and how SLE exposure relates to other family characteristics. These are important questions for future research as it is likely that exposure to family and individual SLEs, cognitive and social/emotional development of the child, parental characteristics and wider sociodemographic factors act in an interactive manner to modulate an individual’s risk for developing mental health difficulties.

Conceptually, results support the proposal by Kerns et al. (2015) that the difficulties in cognitive flexibility often experienced by autistic youth are also a risk factor for the emergence of mental health problems following SLE exposure. This, combined with the increased likelihood of experiencing SLEs associated with autism (Hoover & Kaufman, 2018), may in part explain the high rates of mental health problems in autistic youth. However, we note that although we found a statistically significant pathway from family-SLEs to internalizing problems in autistic children with cognitive flexibility difficulties, the standardized estimates suggest that around 4% of the variance in internalizing symptoms was explained by family-SLEs. Clinically, this not a large effect. This small effect may be in part due to imprecise measurement; more sensitive measures would better estimate the magnitude of associations between the two domains (e.g. using SLE measures that ask specifically about the child’s experiences). Despite the current small effect size, it may still be useful to collect a detailed history of family and individual SLEs in autistic youth with mental health problems, along with information on other potential predictive, protective or mediating factors. Knowledge of precipitating factors (including SLEs) could then guide the choice of support and provide a better-tailored intervention for each individual. There is some evidence to suggest that EF skills can be improved with intervention in autistic children (Kenworthy et al., 2014); however, whether this then buffers against the effects of SLE exposure remains unknown.

This study has several strengths. All existing studies of SLEs in autistic populations are cross-sectional, meaning that directionality cannot be inferred. We use data from repeated yearly assessments of family-level SLEs and child mental health problems over a 4-year period, which generated a fine-grained picture of continuity and change in both family-SLEs and mental health symptoms across childhood in autistic youth. We selected measures rated by different informants to reduce the impact of shared method variance and used a statistical model that took account of trait-like between-person differences in mental health problems. We also undertook additional analyses to test the specificity of effects, which suggested that moderation of the impact of family-SLEs by cognitive flexibility was not solely due to differences in income, autism severity or IQ. However, limitations should be acknowledged. First, the FILE measure used to assess family-SLEs does not give information about the individual impact or severity of different events (i.e. intra-category variability; Dohrenwend, 2006). For example, the death of a relative who has had little contact with a child is far less stressful than the death of a relative who was the child’s primary caregiver (Duggal et al., 2000). Furthermore, this measure does not give any information as to when specific SLEs took place (the measures ask whether SLEs have been experienced by the family unit at any point during the previous 12 months), which could better establish the directionality of associations (and may in part underpin modest effects), and we did not have information about significant SLEs that may have occurred before our first time point of measurement. Interview-based assessment of SLEs that allow more precise timestamping and probing as to the severity of events may overcome these issues (Dohrenwend, 2006). Finally, we chose to use teacher-reported CBCL as our primary outcome to avoid common method variance with parent-reported SLEs (although we report post hoc parent-rated CBCL models in the Supplementary Materials). Teacher report benefits from the wider range of experiences teachers have with similar aged children to inform their ratings. Parents may only draw on experiences with only the target child and their siblings, which has been shown to bias reporting of behaviour (Simonoff et al., 1998). However, it has also been suggested that teachers may be less privy to a child’s internal experiences than parents, therefore potentially leading to less accurate assessment of internalizing symptoms (De Los Reyes & Kazdin, 2005). The current discrepancies in teacher-rated models as compared with parent-rated models highlight the need to consider that different raters may be capturing different aspects of the domain of interest, and researchers should be mindful to these differences when designing studies to better understand autistic mental health. Although self-report is the preferable method to assess internalizing problems, this may be difficult for school-aged children and particularly school-aged autistic children, who are more likely to have difficulties with the identification and communication of internal states. We also used a parent-report measure of cognitive flexibility; replication is required using objective measures of attention/cognitive shifting to understand the role of reporter effects, as scores likely in part reflect parental perception in addition to the true cognitive profile of the child (Vriezen & Pigott, 2002). Finally, despite the strengths of a robust statistical approach and a large and well-characterized longitudinal sample, one cannot assume direct causality. It is still possible that a variable associated with both predictor and outcome could account for the reported association between the two (e.g. genetic factors).

In summary, this study tested bidirectional pathways between family-SLEs and internalizing and externalizing symptoms in a longitudinal, prospective study of autistic children. Results showed that the pathway from family-SLE exposure to future internalizing symptoms was only significant in children with lower cognitive shifting ability. Pathways from internalizing and externalizing symptoms to future family-SLEs were all non-significant. Longitudinal analyses such as those presented currently are crucial to delineate protective or resilience factors for poor mental health in autistic youth. Better knowledge of said factors is key to highlighting intervention targets and therefore promoting positive long-term outcomes.

## Supplemental Material

sj-docx-1-aut-10.1177_13623613211061932 – Supplemental material for Exposure to family stressful life events in autistic children: Longitudinal associations with mental health and the moderating role of cognitive flexibility Click here for additional data file. Supplemental material, sj-docx-1-aut-10.1177_13623613211061932 for Exposure to family stressful life events in autistic children: Longitudinal associations with mental health and the moderating role of cognitive flexibility by Virginia Carter Leno, Nicola Wright, Andrew Pickles, Rachael Bedford, Anat Zaidman-Zait, Connor Kerns, Pat Mirenda, Lonnie Zwaigenbaum, Eric Duku, Teresa Bennett, Stelios Georgiades, Isabel Smith, Tracy Vaillancourt, Peter Szatmari and Mayada Elsabbagh in Autism
