# Supplementary material for: Exposure to family stressful life events in autistic children: Longitudinal associations with mental health and the moderating role of cognitive flexibility
Source: Autism. 2022 Jan 4;26(7):1656–67. doi: 10.1177/13623613211061932 (PMC9483693; doi:10.1177/13623613211061932)
Supplement: sj-docx-1-aut-10.1177_13623613211061932 – Supplemental material for Exposure to family stressful life events in autistic children: Longitudinal associations with mental health and the moderating role of cognitive flexibility [file sj-docx-1-aut-10.1177_13623613211061932.docx]

**Supplementary Materials**

**Table S1. Bivariate Correlations Between Stressful Life Events and Internalizing and Externalizing Symptoms in Autistic Youth**

| **Domain** | **Family-Level SLEs** | | | | **Internalizing Symptoms** | | | | **Externalizing Symptoms** | | | |
| --- | --- | --- | --- | --- | --- | --- | --- | --- | --- | --- | --- | --- |
| **Measure** | **1** | **2** | **3** | **4** | **5** | **6** | **7** | **8** | **9** | **10** | **11** | **12** |
| **FILE T5 (1)** | - |  |  |  |  |  |  |  |  |  |  |  |
| **FILE T6 (2)** | .68^**^ | - |  |  |  |  |  |  |  |  |  |  |
| **FILE T7 (3)** | .60^**^ | .64^**^ | - |  |  |  |  |  |  |  |  |  |
| **FILE T8 (4)** | .54^**^ | .59^**^ | .51^**^ | - |  |  |  |  |  |  |  |  |
| **CBCL INT T5 (5)** | .05 | .08 | .02 | .02 | - |  |  |  |  |  |  |  |
| **CBCL INT T6 (6)** | .10 | .08 | .12 | .06 | .32^**^ | - |  |  |  |  |  |  |
| **CBCL INT T7 (7)** | .22^*^ | -.00 | .15 | .01 | .24^*^ | .36^**^ | - |  |  |  |  |  |
| **CBCL INT T8 (8)** | .19 | .10 | .22^*^ | .11 | .06 | .32^**^ | .42^**^ | - |  |  |  |  |
| **CBCL EXT T5 (9)** | .17^*^ | .11 | .17 | .02 | .41^**^ | .21^*^ | .16 | .18 | - |  |  |  |
| **CBCL EXT T6 (10)** | .02 | .06 | .10 | .07 | .22^*^ | .48^**^ | .17 | .25^*^ | .54^**^ | - |  |  |
| **CBCL EXT T7 (11)** | .17 | .02 | .17 | -.06 | .04 | .26^**^ | .49^**^ | .42^**^ | .51^**^ | .62^**^ | - |  |
| **CBCL EXT T8 (12)** | .06 | .06 | .11 | .03 | -.04 | .15 | .23^*^ | .63^**^ | .40^**^ | .51^**^ | .64^**^ | - |

*p<.05, **p<.01. CBCL= Child Behavior Checklist; FILE=Family Inventory of Life Events and Changes; family-level SLEs= family-level stressful life events. Both FILE and CBCL scores were square-root transformed.

**Table S2. Bivariate Correlations Between Stressful Life Events and Internalizing and Externalizing Symptoms in Autistic Youth with Typical Shifting Ability**

| **Domain** | **Family-Level SLEs** | | | | **Internalizing Symptoms** | | | | **Externalizing Symptoms** | | | |
| --- | --- | --- | --- | --- | --- | --- | --- | --- | --- | --- | --- | --- |
| **Measure** | **1** | **2** | **3** | **4** | **5** | **6** | **7** | **8** | **9** | **10** | **11** | **12** |
| **FILE T5 (1)** | - |  |  |  |  |  |  |  |  |  |  |  |
| **FILE T6 (2)** | .71^**^ | - |  |  |  |  |  |  |  |  |  |  |
| **FILE T7 (3)** | .51^**^ | .60^**^ | - |  |  |  |  |  |  |  |  |  |
| **FILE T8 (4)** | .56^**^ | .57^**^ | .56^**^ | - |  |  |  |  |  |  |  |  |
| **CBCL INT T5 (5)** | .05 | -.03 | .03 | .10 | - |  |  |  |  |  |  |  |
| **CBCL INT T6 (6)** | .16 | .10 | .10 | .10 | .34^*^ | - |  |  |  |  |  |  |
| **CBCL INT T7 (7)** | .16 | .10 | .22 | -.02 | .16 | .28^*^ | - |  |  |  |  |  |
| **CBCL INT T8 (8)** | .03 | .01 | .13 | .12 | .09 | .40^**^ | .30^*^ | - |  |  |  |  |
| **CBCL EXT T5 (9)** | .16 | .13 | .23 | -.02 | .32^**^ | .04 | .13 | .19 | - |  |  |  |
| **CBCL EXT T6 (10)** | -.07 | -.03 | .09 | -.03 | .09 | .39^**^ | .05 | .29^*^ | .38^**^ | - |  |  |
| **CBCL EXT T7 (11)** | .01 | .04 | .24 | -.10 | -.18 | .07 | .42^**^ | .33^*^ | .59^**^ | .60^**^ | - |  |
| **CBCL EXT T8 (12)** | -.10 | -.01 | -.03 | -.11 | -.05 | .17 | .05 | .65^**^ | .45^**^ | .51^**^ | .63^**^ | - |

*p<.05, **p<.01. CBCL= Child Behavior Checklist; FILE=Family Inventory of Life Events and Changes; family-level SLEs= family-level stressful life events. Both FILE and CBCL scores were square-root transformed.

| **Domain** | **Family-Level SLEs** | | | | **Internalizing Symptoms** | | | | **Externalizing Symptoms** | | | |
| --- | --- | --- | --- | --- | --- | --- | --- | --- | --- | --- | --- | --- |
| **Measure** | **1** | **2** | **3** | **4** | **5** | **6** | **7** | **8** | **9** | **10** | **11** | **12** |
| **FILE T5 (1)** | - |  |  |  |  |  |  |  |  |  |  |  |
| **FILE T6 (2)** | .55^**^ | - |  |  |  |  |  |  |  |  |  |  |
| **FILE T7 (3)** | .65^**^ | .63^**^ | - |  |  |  |  |  |  |  |  |  |
| **FILE T8 (4)** | .39^**^ | .53^**^ | .43^**^ | - |  |  |  |  |  |  |  |  |
| **CBCL INT T5 (5)** | -.03 | .14 | -.03 | -.15 | - |  |  |  |  |  |  |  |
| **CBCL INT T6 (6)** | .05 | .13 | .08 | .00 | .38^**^ | - |  |  |  |  |  |  |
| **CBCL INT T7 (7)** | .28 | -.14 | .03 | -.09 | .35^*^ | .43^**^ | - |  |  |  |  |  |
| **CBCL INT T8 (8)** | .29 | .13 | .18 | -.03 | -.01 | .18 | .50^**^ | - |  |  |  |  |
| **CBCL EXT T5 (9)** | .07 | .02 | .03 | -.03 | .44^**^ | .49^**^ | .23 | .23 | - |  |  |  |
| **CBCL EXT T6 (10)** | .01 | .08 | .01 | .13 | .29^*^ | .59^**^ | .26 | .15 | .74^**^ | - |  |  |
| **CBCL EXT T7 (11)** | .30 | -.19 | .03 | -.11 | .29 | .58^**^ | .67^**^ | .41^*^ | .40^**^ | .60^**^ | - |  |
| **CBCL EXT T8 (12)** | .29 | .09 | .10 | .14 | -.08 | .16 | .36^*^ | .59^***^ | .35^*^ | .41^*^ | .61^**^ | - |

**Table S3. Bivariate Correlations Between Stressful Life Events and Internalizing and Externalizing Symptoms in Autistic Youth with Clinically Significant Shifting Problems**

*p<.05, **p<.01. CBCL= Child Behavior Checklist; FILE=Family Inventory of Life Events and Changes; family-level SLEs= family-level stressful life events. Both FILE and CBCL scores were square-root transformed.

**Results from Models Using Parent-Reported Internalizing and Externalizing Symptoms**

*Internalizing Symptoms*

In the full sample, the pathway from internalizing symptoms to family-SLEs was non-significant (b = .01, 95% CIs = -.04 - .06; β = .02, 95% CIs = -.07-.10; p = .70) and therefore dropped from the model. The pathway from family-SLEs to internalizing symptoms was significant (b = -.48, 95% CIs = -.85 - -.12; β = -.31, 95% CIs = -.54 - -.08; p <. 01). Auto-regressive pathways for family-SLEs (b = .86, 95% CIs = .78-.94; β = .96, 95% CIs = .91-.1.04; p < .01) and internalizing symptoms (b = .47, 95% CIs = .07 - .87; β = .46, 95% CIs = .08 - .84; p = .02) were both significant. Cross-sectional correlations between family-SLEs and internalizing symptoms were significant (b = .49, 95% CIs = .34 - .64; β = .77, 95% CIs = .56 - .99; p < .01). Model fit was excellent (χ2(25) = 32.09, p = .16, CFI/TLI = .99, RMSEA = 0.03).

When the sample was split by level of shifting problems, the pathway from family-SLEs to internalizing symptoms was non-significant in the typical (*b* = .02, *95% CIs* = -.65 - .68; *β* = .02, *95% CIs* = -.95 – 1.00; *p* = .96), and the clinically significant shifting problems group (*b* = .02, *95% CIs* = -.82 - .85; *β* = .02, *95% CIs* = -1.06 – 1.05; *p* = .97). The auto-regressive pathway for family-SLEs was significant in both the typical shifting and clinically significant shifting problems groups (*b* = .89, *95% CIs* = .69 – 1.09; *β* = .95, *95% CIs* = .59 - 1.32; *p* < .01; *b* = .84, *95% CIs* = .51 - 1.17; *β* = .91, *95% CIs* = .42 - 1.40; *p* <. 01 respectively). The auto-regressive pathway for internalizing symptoms was significant in the typical (*b* = .68, *95% CIs* = .02 – 1.34; *β* = .59, *95% CIs* = .11 - 1.06; *p* = .04) and clinically significant shifting problems group (*b* = .72, *95% CIs* = .37 – 1.07; *β* = .70, *95% CIs* = .31 - 1.10; *p* < .01). Cross-sectional correlations between family-SLEs and internalizing symptoms were non-significant in both groups (*b* = -.01, *95% CIs* = -.56 - .53; *β* = -.03, *95% CIs* =-1.52 – 1.45; *p* = .96; *b* = -.10, *95% CIs* = -.68 - .49; *β* = -.14, *95% CIs* =-1.00 - .71; *p* = .74). The Wald test of group differences in the family-SLEs to internalizing symptoms path was non-significant (*p* = .99).

*Externalizing Symptoms*

In the full sample, the pathway from externalizing symptoms to family-SLEs was non-significant and therefore dropped from the model *(b* = .03, *95% CIs* = -.11 - .18; *β* = .09, *95% CIs* = -.23 - .40; *p* = .62). The pathway from family-SLEs to externalizing symptoms was also non-significant (*b* = -.05, *95% CIs* = -.91-.82; *β* = -.03, *95% CIs* = -.52-.47; *p* = .91). The auto-regressive pathway was significant for family-SLEs (*b* = .84, *95% CIs* = .75-.93; *β* = .95, *95% CIs* = .85 – 1.06; *p* < .01) and for externalizing symptoms (*b* = .80, *95% CIs* = .55 – 1.04; *β* = .77, *95% CIs* = .53 – 1.01; *p* <.01). Cross-sectional correlations between family-SLEs and externalizing symptoms were non-significant (*b* = .07, *95% CIs* = -.42 - .55; *β* = .20, *95% CIs* = -1.22 – 1.63; *p* = .79). Model fit was excellent (*χ*^2^(25) = 22.21, *p* = .62, CFI/TLI = 1.00, RMSEA = 0.00).

When the sample was split by BRIEF shifting problems, the pathway from family-SLEs to externalizing symptoms was non-significant in the typical shifting (*b* = .39, *95% CIs* = -.48 - .1.25; *β* = .30, *95% CIs* = -.37 - .96; *p* = .38) but significant in the clinically significant shifting problems groups (*b* = .58, *95% CIs* = .17 - .98; *β* = .42, *95% CIs* = .14 - .66; *p* < .01). The auto-regressive pathway for family-SLEs was significant in both groups (*b* = .92, *95% CIs* = .79 – 1.05; *β* = 1.02*, 95% CIs* = .80 - 1.24; *p* < .01; *b* = .92, *95% CIs* = .80 – 1.04; *β* = 1.00, *95% CIs* = .79 - 1.21; *p* < .01 respectively). The auto-regressive pathway for externalizing symptoms was non-significant in the typical shifting (*b* = .67, *95% CIs* = -.34 – 1.68; *β* = .64, *95% CIs* = -.32 – 1.59; *p* = .20) or the clinically significant shifting problems group (*b* = .26, *95% CIs* = -.23 - .74; *β* = .24, *95% CIs* = -.20 - .67; *p* = .30). Cross-sectional correlations between family-SLEs and externalizing symptoms were significant in the typical shifting group (*b* = .60, *95% CIs* = .31 - .89; *β* = .67, *95% CIs* = .14 – 1.19; *p* < .01) and marginally significant in the clinically significant shifting problems groups (*b* = .42, *95% CIs* = -.02 - .86, *β not available, p* = .06). The Wald test of group differences in the family-SLEs to externalizing symptoms path was non-significant (*p* = .59).
